# Target-based fusion using social determinants of health to enhance suicide prediction with electronic health records

**DOI:** 10.1371/journal.pone.0283595

**Published:** 2023-04-26

**Authors:** Shane J. Sacco, Kun Chen, Fei Wang, Robert Aseltine

**Affiliations:** 1 Department of Statistics, University of Connecticut, Storrs, Connecticut, United States of America; 2 Department of Population Health Sciences, Weill Cornell Medicine, New York, New York, United States of America; 3 Division of Behavioral Sciences and Community Health, UConn Health, Farmington, Connecticut, United States of America; University of New South Wales, AUSTRALIA

## Abstract

**Objective:**

Preventing suicide in US youth is of paramount concern, with rates increasing over 50% between 2007 and 2018. Statistical modeling using electronic health records may help identify at-risk youth before a suicide attempt. While electronic health records contain diagnostic information, which are known risk factors, they generally lack or poorly document social determinants (e.g., social support), which are also known risk factors. If statistical models are built incorporating not only diagnostic records, but also social determinants measures, additional at-risk youth may be identified before a suicide attempt.

**Methods:**

Suicide attempts were predicted in hospitalized patients, ages 10–24, from the State of Connecticut’s Hospital Inpatient Discharge Database (HIDD; *N* = 38943). Predictors included demographic information, diagnosis codes, and using a data fusion framework, social determinants features transferred or fused from an external source of survey data, The National Longitudinal Study of Adolescent to Adult Health (Add Health). Social determinant information for each HIDD patient was generated by averaging values from their most similar Add Health individuals (e.g., top 10), based upon matching shared features between datasets (e.g., Pearson’s *r*). Attempts were then modelled using an elastic net logistic regression with both HIDD features and fused Add Health features.

**Results:**

The model including fused social determinants outperformed the conventional model (AUC = 0.83 v. 0.82). Sensitivity and positive predictive values at 90 and 95% specificity were almost 10% higher when including fused features (e.g., sensitivity at 90% specificity = 0.48 v. 0.44). Among social determinants variables, the perception that their mother cares and being non-religious appeared particularly important to performance improvement.

**Discussion:**

This proof-of-concept study showed that incorporating social determinants measures from an external survey database could improve prediction of youth suicide risk from clinical data using a data fusion framework. While social determinant data directly from patients might be ideal, estimating these characteristics via data fusion avoids the task of data collection, which is generally time-consuming, expensive, and suffers from non-compliance.

## Introduction

Suicide is the second most common cause of death among adolescents and young adults [1]. Recent data from the CDC indicate that death by suicide among children and young adults ages 10–24 in the US increased 57% between 2007–2018, from 6.8 to 10.7 per 100,000 [2]. There is substantial evidence that both adults and youth at risk of suicide are in contact with the healthcare system, yet providers are often unaware of their risk [3]. To address this problem, the National Institute of Mental Health has prioritized the development of suicide prediction algorithms for use in clinical settings [4]. As a result of these efforts, there are now a number of published algorithms using data mining and machine learning approaches with clinical data to predict suicidal behavior and suicide mortality among patients in large healthcare systems [5–7]. Such studies have not only confirmed the importance of prominent clinical risk factors for suicide attempts and death identified in prior research [8], but have also identified a myriad of other characteristics predictive of suicidal behavior, resulting in greatly improved predictive accuracy compared to previous efforts (e.g., social isolation [9]).

Although less numerous, algorithms predicting suicide risk among children and adolescents have also shown promise. Using machine learning models to predict suicide attempts among 10–18 year old patients in a large pediatric medical center, Su et al [6] detected 65% of cases given 90% specificity. In a similar study using a logistic regression approach to predict suicide attempts in 10–24 year old patients hospitalized in Connecticut, Xu et al [5] detected a slightly lower percent of cases (43%) at 90% specificity. In another study using a naïve Bayes classifier approach to predict suicide attempts among patients in a large medical network including a pediatric hospital, Barak-Corren et al [10] detected 37% of cases at 90% specificity. Taken together such efforts indicate that risk factors gleaned from patient medical records–demographic characteristics, diagnosis codes, medications, and healthcare utilization patterns (e.g., ED visits)–may identify 37% to 64% of cases when deploying predictive algorithms with setting specificity to 90%.

One possible method of improving these results may be found by expanding the range of predictors included in the model. There is a vast literature indicating that factors such as social support, stressful life events, and expectations of the future are strongly associated with mental health generally and suicide-related thoughts and behaviors in particular [11–14]. These risk factors may broadly be referred to as social determinants of health, describing the conditions and environments in which individuals are born and live [15]. Social determinants may be extracted from structured and unstructured EHR data; as shown in several recent studies [16, 17], prediction of suicide could be improved by incorporating social determinant information extracted from clinical notes through natural language processing (NLP). However, clinical notes data may not be always available for predictive modeling purposes, and even if they are, NLP processing could be very costly. Moreover, EHR data may be insufficient to capture a complete and accurate set of social determinants; while several environmental and social domains such as housing, social support, and financial conditions may be extracted from EHRs, many social and behavioral determinants that impact health could be missed [18]. Ideally, social determinants may be assessed with more thoroughness via well-designed and validated questionnaires, but they are costly to collect during healthcare encounters, and are subject to high rates of non-compliance [19].

Fortunately, such questionnaire data are commonly collected in large-scale studies that survey health and well-being. The National Longitudinal Study of Adolescent to Adult Health (Add Health) collects a substantial amount of information about social determinants and related concepts, such as parental relationship quality, peer interactions, and perceptions of town safety, as well as medical information similar to the information available in the EHR, such as a history of depression, substance abuse, etc. [20].

One novel means of accessing social determinant information for suicide risk prediction with EHR is to harvest this data from other data sources such as Add Health and incorporating this information in predictive modeling via principles of *transfer learning*. In this case, transfer learning refers to increasing knowledge about a task (e.g., suicide prediction) in one dataset with knowledge from another [21], in particular, transferring task-related features from one dataset to another lacking said task-related features. Although the survey data and clinical records are collected independently, similarities among individuals in the survey and clinical datasets may allow us to “borrow” social determinant information for prediction.

In this retrospective study, built upon an initial investigation by Xu et al [5], we apply and generalize a transfer learning framework to incorporate social determinant of health measures that serve as risk factors for suicidal behavior in predictive models using clinical data from inpatient hospitalizations. Specifically, as a proof-of-concept, data fusion techniques will be used to fuse social determinants found important to suicide risk prediction in Add Health to the State of Connecticut’s Hospital Inpatient Discharge Database (HIDD), including only hospital inpatient EHR data. First, we aim to determine if including social determinant of health features in HIDD models improves model performance versus using clinical data only. Second, if performance is improved, we describe the top predictors in the model as well as characteristics of newly identified cases compared to the clinical model. Finally, we aim to highlight social determinant of health measures that uniquely predict suicide attempts.

## Method

This study was approved by the University of Connecticut Health Center Institutional Review Board, Weill Cornell Medical College Institutional Review Board, and the CT Department of Public Health Human Investigations Committee.

### Datasets and cohorts

#### Hospital Inpatient Discharge Database

The Hospital Inpatient Discharge Database (HIDD) was our primary (or target) dataset for suicide risk modeling. As the only HIPAA identifiers were dates of birth, dates of service, and 5-digit zip code, a waiver of informed consent was granted. The HIDD consists of hospital inpatient demographic information and International Classification of Diseases (ICD) medical codes from Connecticut commercial claims for patients from January 1, 2012 to September 30, 2017. Since both ICD-9 and 10 codes were present, codes were unified to ICD-9 using the package *touch* in R [22] for a coherent feature set. We define suicide attempts by the presence of any intentional self-harm codes (ICD-9 E950.XX-E958.XX) or the presence of suicidal ideation (ICD-9 V62.84) or mental disorders with potential of the same (e.g., ICD-9 296.XX episodic mood disorder) and other diagnosis codes indicative of self-harm (see S1 Table for details) [5, 23]. The first suicide attempt following at least one non-attempt visit was our primary outcome. ICD-9 codes defining attempts above were then removed and the remaining codes were examined as predictors of attempts. Given the vast number of ICD-9 codes, five-digit predictor codes were aggregated based upon their higher-level, three-digit category for analyses. The presence of predictor codes from non-event visits were coded, and if an attempt occurred, only predictor codes *prior* to the first event visit were coded. This was subsequently our window for prediction (i.e., last non-event visit to event visit within the data collection window of 2012–2017). No recruitment window was defined (i.e., all patients were eligible for inclusion).

The HIDD cohort included patients between the ages 10 to 24, of which 484 had a suicide attempt during the measurement period (cases), and 38459 did not (controls).

#### The National Longitudinal Study of Adolescent to Adult Health

The National Longitudinal Study of Adolescent to Adult Health (Add Health) was our external source for social determinant information and consisted of self-reported survey responses from children to young adults across the United States since January 1994 [20]. This dataset was accessed in an anonymized format and is publicly available at the Inter-University Consortium for Political and Social Research (ICPSR) website. Participant written consent and/or assent was obtained by the original study investigators. Suicide risk was assessed via suicidal ideation and attempt frequency items, similar to other surveys (e.g., Youth Risk Behavior Surveillance Survey [24, 25]). Specifically, participants were asked, “*During the past 12 months*, *did you ever seriously think about committing suicide*?” If “*Yes*”, they were then asked, *“During the past 12 months*, *how many times did you actually attempt suicide*?” If a participant said “*1 time*” or more to the second item, they were coded as cases. In our study, the Add Health cohort was children to young adults, ages 10 to 20 during the baseline wave of the subject in 1994, and there were 230 cases and 6271 controls.

The Add Health data included a wide net of social determinants and related features that are known suicide risk factors [11–14, 26]. We considered 23 features. This included (1) core social support features (number of siblings, perception that their father cares, perception that their mother cares, and weekly frequency of hanging out with friends). Features also included (2) social interaction and related concepts (daily hours driving, daily hours being online, daily hours watching TV, employment status, English grades, math grades, having ever had intercourse, STD risk, and religious orientation as being non-religious, Christian, Catholic, or Baptist). As well, features included (3) related thoughts and behaviors (annual frequency of physical altercations while intoxicated, body image, eats no breakfast, and needing medical attention but not seeking treatment), and (4) present and expected future environment features (anticipation of being killed by age 21, desire to attend college, lives in one-family house, happy living in their town, and perceived town safety). Most of the features had <5% missingness (included *M* = 1.2%, *CI*_*95%*_ = [0.6%, 1.8%]) and missing values of each feature were replaced by its median. See S2 Table for more details.

### Statistical analysis

#### Data fusion framework

To utilize social determinant information from Add Health in HIDD for predictive modeling, we applied and generalized the transfer-learning based data fusion framework found in Xu et al [5]. This generalized framework, in the context of the present study, included four major steps: (1) linking HIDD and Add Health via creation of mock ICD codes, (2) identifying and screening suicide risk of social determinant of health features in Add Health, (3) computation of suicide-specific patient similarity measures, and transferring selected Add Health features to HIDD via similarity-weighted data aggregation, and (4) performing predictive modeling in the HIDD with both HIDD features and transferred information from Add Health. More details are provided as follows.

*Step 1*: *Linking Add Health to HIDD*. In this step, a set of shared features was identified or created to link Add Health and the HIDD. Add Health and HIDD did not contain directly overlapping features aside from age and gender. To possibly match and integrate the two datasets, response items in Add Health related to medical diagnoses were used to create mock ICD-9 codes. These included items assessing the absence/presence of a range of conditions (e.g., asthma, diabetes, STDs), and symptomatology (e.g., acne, alcohol dependence, depression, muscle and joint pain). See S3 Table for item and coding details.
*Step 2*: *Identify and screen suicide-related features in Add Health*. In this step, social determinant features in Add Health that were most relevant to suicide risk were identified and screened, in preparation to be transferred to the HIDD.

First, each mock ICD-9 code and social determinant of health feature were marginally screened for suicide risk prediction using logistic regression, controlling for age and gender. Features with *p-*values < .10, adjusted for false discovery, were then examined for joint prediction of suicide risk via regularized elastic net logistic models with cross-validation for model selection. All predictor features were normalized (i.e., transformed to a 0–1 scale). To stabilize the selection results and assess out-of-sample performance, the data was split 9–1 training-validation and the above process was repeated 10 times. Any social determinant features that were selected at least 50% of times were kept for the next step.

*Step 3*: *Transferring Add Health features to HIDD*. In this step, pairwise patient similarity measures were computed from the shared features, and new HIDD features were generated based upon similarity measures.

To quantify the similarity between HIDD patients and Add Health participants, weighted Pearson’s correlation coefficients and weighted Manhattan distances were computed from the shared features, as well as, their relative importance values in suicide risk prediction (based on a joint suicide-risk model with age, gender, and overlapping ICD-9 codes using the Add Health data). As such, for each patient in HIDD, we obtained their suicide-specific patient similarities with each of the participants in Add Health. The weighted similarity measures generalize those unweighted versions used in Xu et al [5]; see S1 File for details on the weighted similarity measures.

New HIDD features were then created utilizing selected Add Health social determinant data from Step 2 and similarity scores computed in this step. For each HIDD patient, *i* = 1, …, *n*^*t*^ the total sample size of HIDD, a single fused value ${\text{P}}_{\text{i}}^{\text{t}}$ was generated by averaging the values of their respective *k* top-ranked Add Health matches for each social determinant feature. A grid of *k* top Add Health matches were included, where *k* = {10, 20, 50, 100, and 200}.

*Step 4*: *Predictive modeling in HIDD*. In the HIDD, suicide attempts were modelled based on either age, gender, and ICD-9 diagnosis codes alone (conventional model), or by additionally utilizing fused social determinant features such as the average value of body image from the top 20 most similar Add Health matches using Manhattan distance (fusion model). Attempts were also modelled by age, gender, and fused social determinants alone for comparison with the conventional and fusion models. We followed the same “marginal screening + regularized logistic modeling” procedures as Step 2 and past related works [5, 27]. Here, fusion features were screened also controlling for age, gender, and screened ICD-9 codes.

We stress that while we use the same target dataset and suicide outcome measures as in Xu et al [5], the current study is distinct and novel in several aspects, including that (1) we focus on borrowing information from health survey data rather than from other clinical data, (2) we propose to transfer important social determinants features rather than overall estimated risk scores, and (3) we generalize the framework in Xu et al [5] by allowing mock matching and considering weighted suicide-specific similarity measures.

#### Model evaluation and feature importance

To evaluate model performance, multiple random splitting procedures were applied (90% training and 10% testing), and the out-of-sample area under the curve (AUC) as well as the sensitivity and positive predictive value (PPV) at 90 and 95% specificity were calculated and averaged over 10 splits. For each model, features selected as predictors were described and compared in terms of their selection frequency and estimated effect size (as measured by average coefficient magnitude).

To describe which additional cases were detected by the fusion versus conventional model, all features were compared between true and false prediction groups by model. The cut-off for predicted classification was set to the top 10% highest risk scores in models. ANOVA and chi-square tests were utilized as appropriate. For features that differed by group overall at *p-*value < .05, multiple comparisons were executed for conventional model only and fusion model only true prediction groups. Given there were 20 versions of fused scores for each social determinant feature (i.e., 4 similarity measures by 5 top-*k* ranks), these comparisons were adjusted for familywise error via Bonferroni correction (*p-*value *= p-*value/*k* related features = .05/20 = .0025).

Lastly, to capture which individual social determinant features may have predicted suicide attempts, features were ranked by marginal importance to attempt prediction in the full data as well as selection frequency in resampled fusion models. Marginal hypothesis testing was carried out in the full data where each fusion feature was entered into a logistic regression model, controlling age, gender, and screened ICD-9 codes. Results were ranked by *p*-values adjusted for false discovery, then absolute coefficient magnitude, and lastly selection frequency. The top-performing similarity method and rank version of each fused social determinant feature was reported.

## Results

HIDD and Add Health cohorts were described and summarized in Table 1. HIDD and Add Health cases were a majority female, and within the 15–19 age group. HIDD had more cases in the 20–24 age group while Add Health had more cases in the 10–14 age group; there were zero cases in the 20–24 Add Health age group. In Add Health, 3.5% of participants reported a prior suicide attempt, more than double the rate of adolescents and young adults requiring inpatient hospitalization for a suicide attempt in the HIDD (1.2%).

**Table 1:** HIDD and Add Health cohort descriptions.

Variable	HIDD		Add Health	
	Case	Control	Case	Control
Sample size, *N* (%)	484 (1.24)	38459 (98.76)	230 (3.54)	6271 (96.46)
Sex, *N* (%)				
Female	307 (63.43)	22895 (59.53)	160 (69.57)	3194 (50.93)
Male	177 (36.57)	15564 (40.46)	70 (30.43)	3077 (49.07)
Age group^†^ , *N* (%)				
10–14	72 (14.88)	6626 (17.23)	110 (47.83)	2935 (46.80)
15–19	235 (48.55)	12679 (32.97)	120 (52.17)	3324 (53.01)
20–24	177 (36.57)	19154 (49.80)	0 (00.00)	12 (00.19)

Note. HIDD = Hospital Inpatient Discharge Database.

^†^Age reported in 2014 and 1994 for HIDD and Add Health, respectively.

### Screening social determinant of health features in Add Health

The importance of social determinant features to suicide risk in Add Health was assessed via statistical modeling. Twelve of twenty-three social determinant features appeared as model predictors in at least 50% of times during the resampling procedure. This included anticipating being killed by age 21, being non-religious, body image, desire to attend college, eating no breakfast, English grades, had ever had intercourse, happy living in their town, needing medical attention but not seeking treatment, perceived town safety, and perception that their mother cares.

### Predictive modeling in HIDD

**Fig 1:**
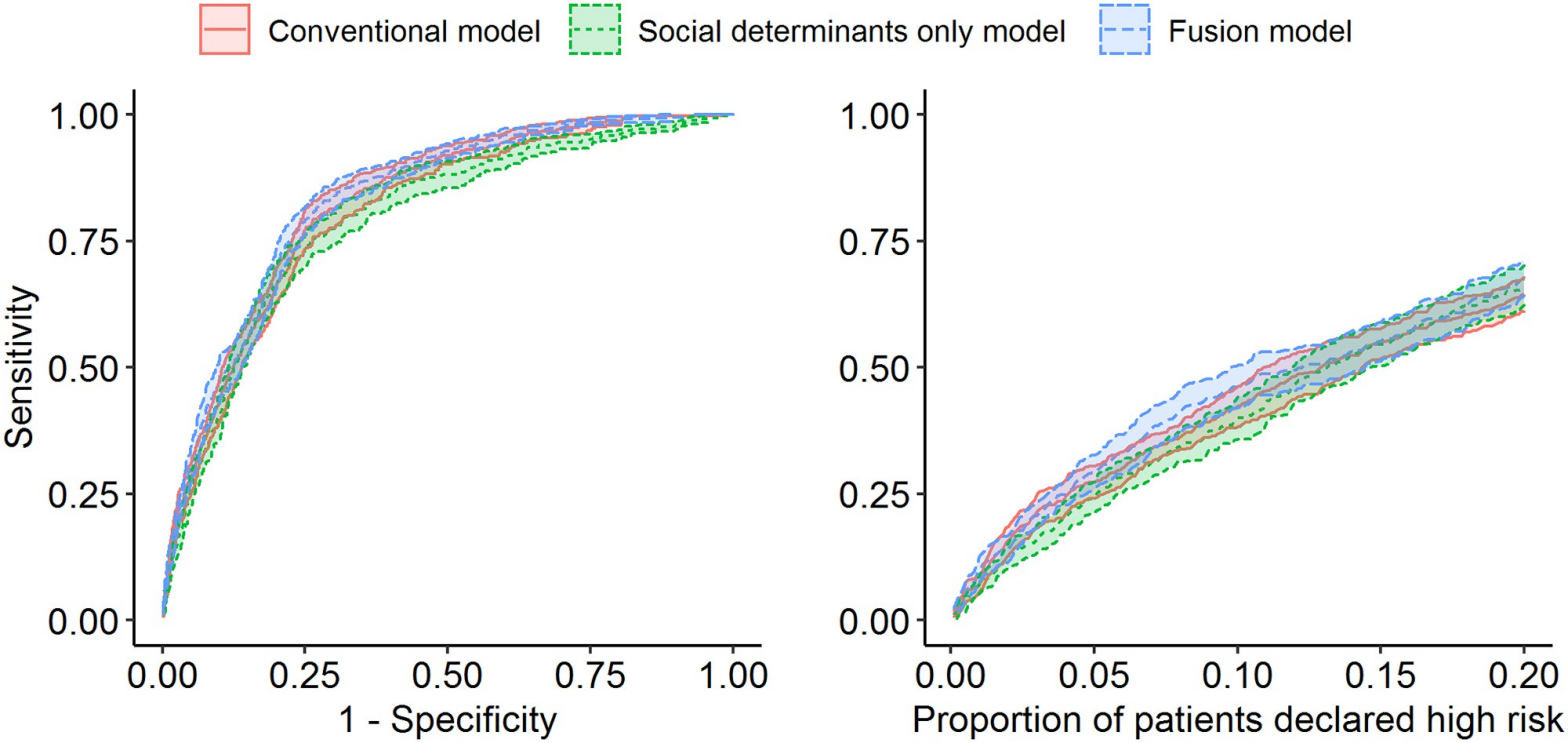
ROC curve and proportion cut-off for classification by sensitivity plot comparing resampled conventional, social determinant of health only, and fusion models.

Results of the convention model, the social determinants only model, and the fusion model for predicting suicide attempts with HIDD data were summarized in Table 2 and visualized in Fig 1. The fusion model provided the best performance across different metrics, followed by the conventional model and the social determinants only model, in that order. On average, the fusion model provided almost a 10% improvement in sensitivity and PPV and a 1.3% improvement in AUC compared to the conventional model; there was approximately a 15–20% and 5% improvement in the same, compared to the social determinants only model, respectively. There were an average of 50 cases in validation sets (*CI*_*95%*_ = [45, 55]), and when setting classification cut-offs to the top 10% of modelled risk, the fusion model predicted 46% of cases on average (*M* = 23, *CI*_*95%*_ = [20, 27]), the conventional model predicted 42% (*M* = 21, *CI*_*95%*_ = [18, 24]), and the social determinants only model predicted 40% (*M* = 20, *CI*_*95%*_ = [18, 22]).

**Table 2:** Performance of the conventional, social determinant of health only, and fusion models using HIDD data. Reported are averaged out-of-sample performance measures and their associated 95% confidence intervals from the repeated random splitting procedure.

Model	AUC, *M* (*95% CI*)	Sensitivity, *M* (*95% CI*)		Positive predictive value, *M* (*95% CI*)	
		95% specificity	90% specificity	95% specificity	90% specificity
Conventional model	0.82 (0.81, 0.83)	0.28 (0.25, 0.31)	0.44 (0.39, 0.49)	0.068 (0.059, 0.077)	0.054 (0.046, 0.063)
Social determinants only model	0.79 (0.78, 0.81)	0.26 (0.22, 0.30)	0.41 (0.36, 0.46)	0.062 (0.054, 0.071)	0.050 (0.044, 0.055)
Fusion model	0.83 (0.82, 0.84)	0.31 (0.27, 0.35)	0.48 (0.43, 0.52)	0.074 (0.064, 0.085)	0.058 (0.050, 0.067)
Fusion—conventional model	0.01 (<0.01, 0.02)	0.03 (<0.01, 0.05)	0.04 (0.01, 0.06)	0.007 (0.001, 0.013)	0.004 (0.002, 0.006)

Note. AUC = area under the curve. CI = confidence interval.

Predictor selections were then examined and compared. For the conventional model, there were 887 candidate features, among which 52 appeared as predictors in at least one resampled model and 22 appeared in all resamples. These predictors included age, gender, mental health disorders and symptoms (e.g., acute stress reaction, episodic mood disorders), poisoning by various substances (e.g., analgesics, anticonvulsants), immune disorders (e.g., allergies, anemias), and local social determinant codes (e.g., outcome of delivery, unemployment).

For the social determinants only model, there were 240 candidate features from 12 social determinants of health, among which 122 appeared as predictors in at least one resampled model and 19 appeared in all resamples. These predictors included age and all social determinant types (e.g., being non-religious, perception that their mother cares), except for body image. Three versions of needing medical attention but not seeking treatment appeared given different similarity methods and top-ranked matches included. Two versions of anticipation of being killed by age 21, desire to attend college, eating no breakfast, happy living in their town, and perception that their mother cares appeared.

The predictors selected by the fusion model (those that appeared in all resamples) were summarized in Table 3. Of the 1127 candidate features (887 conventional features and 240 fused features), 113 appeared as predictors in at least one resampled model. Twenty-six predictors appeared in all resamples. Nineteen of these predictors were from the conventional model; however, gender, nondependent drug abuse, and personal history of health hazards (e.g., allergies) were excluded. Four of these predictors were from the social determinant only model, including single versions (based upon similarity method and top-ranked matches included) of being non-religious, desire to attend college, perceived town safety, and perception that their mother cares. Three additional social determinants of health also appeared as predictors in all resamples, including body image, having physical altercations while intoxicated, and a second version of being non-religious based upon similarity method and top-ranked matches included. The top five predictors in fusion models by selection frequency and absolute coefficient magnitude were three risk factors: having a poisoning by hormone substitutes diagnosis, poisoning by anticonvulsants diagnosis, and an acute stress reaction diagnosis, and two protective factors: being older and being non-religious.

**Table 3:** Predictors selected by all fusion models in the repeated random-splitting procedure.

Predictors	Description		Model coefficients
	Cases	Controls	Odds, *M* (*SD*)
No of patients, *N* (%)			
	484 (1.24)	38459 (98.76)	-
Age, *M* (*SD*)			
	17.83 (3.44)	19.12 (4.41)	0.07 (1.33)
ICD-9 Medical diagnoses, *N* (%)			
*962*, *Poisoning by hormone substitutes*	≤6 (≤1.24)*	≤6 (≤0.02)*	15.50 (1.12)
*966*, *Poisoning by anticonvulsants*	≤6 (≤1.24)*	12 (0.03)	4.18 (1.15)
*308*, *Acute reaction to stress*	≤6 (≤1.24)*	60 (0.16)	3.97 (1.16)
*965*, *Poisoning by analgesics*	17 (3.51)	207 (0.54)	3.16 (1.13)
*296*, *Episodic mood disorders*	333 (68.80)	8163 (21.23)	3.07 (1.09)
*V63*, *Other medical facilities unavailable*	≤6 (≤1.24)*	25 (0.07)	2.01 (1.25)
*V27*, *Outcome of delivery*	14 (2.89)	8429 (21.92)	0.56 (1.10)
*V62*, *Unemployment*	210 (43.39)	3899 (10.14)	1.82 (1.03)
*307*, *Special symptoms/syndromes NOS*	47 (9.71)	841 (2.19)	1.55 (1.05)
*V60*, *Housing/economic circumstances*	17 (3.51)	271 (0.70)	1.61 (1.09)
*301*, *Personality disorders*	69 (14.26)	1080 (2.81)	1.50 (1.05)
*311*, *Depressive disorders NOS*	128 (26.45)	3581 (9.31)	1.51 (1.03)
*969*, *Poisoning by psychotropic agents*	11 (2.27)	152 (0.40)	1.46 (1.20)
*300*, *Anxiety disorders*	205 (42.36)	5758 (14.97)	1.38 (1.03)
*309*, *Adjustment reaction*	93 (19.21)	2067 (5.37)	1.24 (1.08)
*304*, *Drug dependence*	54 (11.16)	1341 (3.49)	1.27 (1.05)
*V61*, *Other family circumstances*	67 (13.84)	1182 (3.07)	1.14 (1.05)
*285*, *Other & unspecified anemias*	11 (2.27)	2584 (6.72)	0.92 (1.08)
Fusion features (similarity method, top rank), *M* (*SD*)			
*Being non-religious (d*, *20)*	0.09 (0.04)	0.10 (0.05)	0.30 (1.33)
*Body image (dw*, *10)*	3.39 (0.28)	3.43 (0.34)	2.45 (1.51)
*Perception that their mother cares (rw*, *50)*	4.81 (0.08)	4.86 (0.07)	0.44 (1.19)
*Being non-religious (r*, *100)*	0.13 (0.04)	0.12 (0.03)	1.76 (1.36)
*Desire to attend college (r*, *200)*	4.02 (0.23)	4.10 (0.20)	0.40 (1.26)
*Physical altercations while intoxicated (dw*, *50)*	0.00 (0.01)	0.00 (0.01)	0.62 (1.34)
*Perceived town safety (r*, *20)*	0.82 (0.08)	0.87 (0.07)	0.73 (1.15)

Note. ICD = International Classification of Diseases. d = unweighted Manhattan distance. dw = weighted Manhattan distance. r = unweighted Pearson’s correlation coefficient. rw = weighted Pearson’s correlation coefficient. SD = standard deviation.

*Due to Connecticut Department of Public Health requirements, frequencies n≤6 cannot be disclosed.

Differences in features between cases picked up exclusively by the conventional or fusion models using the full data were summarized in Table 4. Identified cases differed only by fusion features (all types except for body image). The top five differences by absolute percent difference magnitude were fusion model only cases had ever had intercourse less, had physical altercations while intoxicated less, needed medical attention but did not seek treatment less, ate no breakfast less, and were non-religious less.

**Table 4:** Comparison of identified cases by conventional and fusion models.

Features	Similarity method	Rank included	Both Models	Conventional model only	Fusion model Only	Fusion–conventional model	Neither model
No of patients, *N* (%)	-	-	211 (43.60)	27 (5.58)	42 (8.68)	15	204 (42.15)
Fusion features, *M* (*SD*)							
*Desire to attend college*	*d*	100	4.26 (0.12)	4.18 (0.18)	4.30 (0.08)	0.12	4.28 (0.12)
Eats no breakfast*	*d*	20	0.12 (0.08)	0.17 (0.11)	0.08 (0.07)	-0.09	0.10 (0.10)
*English grades*	*dw*	10	2.00 (0.26)	2.22 (0.26)	1.85 (0.19)	-0.37	1.92 (0.24)
Physical altercations while intoxicated*	*rw*	10	0.02 (0.04)	0.07 (0.06)	0.01 (0.02)	-0.06	0.02 (0.03)
Happy living in their town*	*d*	20	3.79 (0.40)	3.74 (0.44)	4.03 (0.23)	0.29	4.10 (0.34)
Ever had Intercourse*	*dw*	50	0.21 (0.19)	0.30 (0.23)	0.10 (0.03)	-0.20	0.14 (0.15)
Anticipation of being killed by 21*	*r*	10	1.69 (0.26)	1.54 (0.21)	1.77 (0.23)	0.23	1.71 (0.27)
Perception that their mother cares*	*rw*	10	4.83 (0.18)	4.78 (0.16)	4.89 (0.12)	0.11	4.87 (0.12)
Needed medical attention but did not seek treatment*	*dw*	10	0.16 (0.17)	0.21 (0.21)	0.08 (0.10)	-0.13	0.13 (0.13)
Being non-religious*	*d*	20	0.09 (0.04)	0.13 (0.07)	0.07 (0.02)	-0.06	0.09 (0.04)
*Perceived town safety*	*rw*	10	0.78 (0.12)	0.88 (0.06)	0.76 (0.13)	-0.12	0.86 (0.10)

Note.

*Multiple versions of the feature appeared and only the version with largest fusion-conventional model difference was shown.

d = unweighted Manhattan distance. dw = weighted Manhattan distance. r = unweighted Pearson’s correlation coefficient. rw = weighted Pearson’s correlation coefficient. SD = standard deviation.

Finally, the degree to which individual social determinant features predicted suicide attempts was examined and reported in Table 5. In regard to marginal testing using the full HIDD data, each type of social determinant predicted attempts. Based upon ranking marginal test results and selection frequency in resampled fusions models, the top five predictors were three risk factors: having an overweight body image, worse English grades, and being non-religious, and two protective factors: stronger perception that their mother cares and desire to attend college.

**Table 5:** Marginal testing of fused social determinant of health features in the full HIDD data and selection frequency in resampled fusion models.

Fusion features	Resampled fusion model		Marginal hypothesis testing in full HIDD data		
	Top similarity method (Rank)	Selected, *N*	Top similarity method (Rank)	Log odds (*SE*)	*p*-value
Body image	*dw* (10)	10	*dw* (20)	6.70 (0.47)	<.001
Perception that their mother cares	*rw* (50)	10	*rw* (100)	-5.54 (0.31)	<.001
Perceived town safety	*r* (20)	10	*r* (20)	-4.25 (0.27)	<.001
Desire to attend college	*r* (200)	10	*r* (50)	-5.50 (0.44)	<.001
Being non-religious	*d* (20)	10	*dw* (20)	3.28 (0.31)	<.001
Physical altercations while intoxicated	*dw* (50)	10	*dw* (20)	2.86 (0.47)	<.001
Needed medical attention but did not seek treatment	*d* (100)	8	*rw* (200)	3.08 (0.32)	<.001
English grades	*r* (10)	6	*dw* (50)	4.49 (0.38)	<.001
Happy living in their town	*d* (100)	5	*r* (10)	-3.86 (0.29)	<.001
Anticipation of being killed by age 21	*rw* (200)	4	*dw* (20)	7.67 (0.41)	<.001
Ever had intercourse	*d* (200)	7	*d* (200)	-1.39 (0.21)	<.001
Eats no breakfast	*d* (20)	5	*d* (20)	-2.70 (0.33)	<.001

Note. HIDD = Hospital Inpatient Discharge Database. d = unweighted Manhattan distance. dw = weighted Manhattan distance. r = unweighted Pearson’s correlation coefficient. rw = weighted Pearson’s correlation coefficient. SE = standard error.

## Discussion

This study is the first to apply a data fusion framework to incorporate social determinants of health from survey data when modeling suicide attempts with EHR data, and provides subsequent proof-of-concept for prediction improvement via data fusion beyond Xu et al [5]. We found that many measures of social determinants were associated suicide risk in the Add Health survey data, and when said features such as being non-religious or perception that their mother cares were included as fusion features in HIDD, they improved prediction compared to the conventional model. Specifically, the fusion model improved the identification of true positive patients by almost 10% at 90 and 95% specificity compared to the conventional model. These improvements support past literature suggesting that these aspects of social determinants of health may be unique risk factors for suicide (e.g., [11, 12]). While improvements were modest, this investigation provides proof of concept that the fusion of social determinants of health measures from survey data with clinical data may improve the prediction of suicide attempts based solely on medical records.

This finding is especially important given the recent emphasis on collecting patient-reported outcome data, including social determinants of health information [28] and self-reports of suicidal thoughts and behaviors, by healthcare providers and health systems (e.g., Zero Suicide Initiative [29]). Although a handful of studies suggest collection of such data may be feasible (e.g., [30, 31]), some argue otherwise due to issues such as staff non-compliance or insufficient funding (e.g., [19]). For instance, Sisodia et al [32] reported that over the four years of an initiative to collect patient-reported outcomes within a large healthcare network in Massachusetts, data were missing at 50% of examined facilities and/or more than 50% incomplete. While primary data directly from patients may be ideal, estimating suicide risk as well as social determinants in patients via data fusion, as opposed to collecting this information in-person, may provide a cost-effective solution to risk identification for healthcare systems.

In regards to *how* fusion improved the conventional model, social determinants of health appeared to provide direct contributions to risk prediction. Firstly, six social determinants consistently improved prediction beyond conventional predictors selected (i.e., being non-religious, body image, perception that their mother cares, desire to attend college, physical altercations while intoxicated, and perceived town safety). Given conventional predictors included prominent risk factors (e.g., episodic mood disorders), social determinants likely provided novel information about risk. This is opposed to refining knowledge about conventional predictors, alike Xu et al [5], which fused calculated risks from more comprehensive EHR data. Secondly, when comparing the identification of cases between conventional and fusion models, identified cases differed only by fusion features, suggesting model differences likely derived from fusion features and not conventional ones. This opposes the presence of differences in conventional features, which would convey knowledge about said features changed between models, as in Xu et al [5], where identified cases between models differed on thirteen conventional features. Lastly, the social determinant only model performed reasonably well (AUC = 0.79), supporting the notion that these features may serve as unique risk factors, even as estimated by data fusion. This is a stark contrast to Xu at al [5] where fused risk scores alone displayed poor performance, being near chance (AUC = 0.52). The above model characteristics and differences, especially when compared to Xu et al [5], appear to indicate that data fusion could improve prediction through at least two methods: direct contributions and coefficient refinement, respectively.

Interestingly, fused social determinant features with various similarity methods, weighting schemes, and top-ranked matches included were selected in the fusion model, conveying not only that various social determinants aided prediction but also that use of multiple approaches to generating fusion features may have been important. Specifically, not all methods of matching patients on similarities are equivalent insofar as the characteristics of generated fusion features in predicting risk (e.g., multiple versions of being non-religious based upon similarity method were jointly important in prediction). To explain this phenomenon further, additional investigation is needed, exploring such differences in similarity methods and prediction among the related fusion features.

The present study had limitations. There were few Add Health participants in the 20–24 age group. Subsequently, similarity matches for 20–24 HIDD patients may have been less alike than those available for 10–19 HIDD patients. If additional 20–24 Add Health participants were available model performance and improvements may have differed, as we anticipate that the fusion approach could be even more powerful when survey data with similar demographics are available and utilized. In our study, HIDD data did not contain much social determinants information itself; as such, it would be interesting to apply our methods with more comprehensive EHR data to investigate whether fusion from external health survey data with rich social determinants information remains beneficial when EHR derived social determinants features are included. Moreover, since this study utilized nonclinical survey data from Add Health to estimate social determinants, future studies may benefit from using validated measures from more clinically-oriented data to estimate social determinants (e.g., The National Survey on Drug Use and Health [33]), as improvements due to fusion may have differed or been stronger within this application.

The current study displayed proof of concept that survey data, in particular social determinants of health, may enhance prediction compared to medical codes alone. In particular, when estimating and including social determinant concepts important to suicide risk such as social support and expectations of the future via data fusion, models also containing many diagnoses important to suicide risk had increased predictive performance across all assessed metrics. Improving suicide risk prediction via social determinants and data fusion may help identify and prevent additional suicides compared to the present state-of-the-art methods if implemented in healthcare settings. Our study is one step in a broader effort to leverage multiple external data sources in suicide risk modeling, with hopes of eventually deploying these models in clinical support platforms that assist healthcare professionals in assessing suicide risk.

## Supporting information

S1 Table Suicide event coding in HIDD. (PDF)

S2 Table Table of missingness, ranges, and medians of social determinant of health features in Add Health. (PDF)

S3 Table Add Health coding form for similarity matching with HIDD. (PDF)

S1 File Similarity matching between HIDD and Add Health data. (PDF)
