# Supplementary material for: Target-based fusion using social determinants of health to enhance suicide prediction with electronic health records
Source: PLoS One. 2023 Apr 26;18(4):e0283595. doi: 10.1371/journal.pone.0283595 (PMC10132649; doi:10.1371/journal.pone.0283595)
Supplement: S1 Table — (PDF) [file pone.0283595.s001.pdf]

S1 Table. Suicide event coding in HIDD.

Code type	ICD-9 codes
Intentional self-harm	E950.XX-E958.XX
Suicidal ideation & open wounds or poisoning in same visit	V62.84
	and
	870.XX-899.XX or 960.XX-989.XX
Mental health disorders & open wounds or poisoning indicative of self-harm in same visit	280.8-290.9, 293.83, 295.XX, 296.00-296.06, 296.1-296.14, 296.20-296.36, 296.40-296.99, 297.XX, 298.0-298.9, 299.XX, 300.4, 301.XX, 309.0-309.9, 311.XX, 780.1
	and
	881.XX, 960.XX-989.XX, or 994.7

Notes. ICD=International Classification of Diseases.
