# Supplementary material for: Target-based fusion using social determinants of health to enhance suicide prediction with electronic health records
Source: PLoS One. 2023 Apr 26;18(4):e0283595. doi: 10.1371/journal.pone.0283595 (PMC10132649; doi:10.1371/journal.pone.0283595)
Supplement: S2 Table — (PDF) [file pone.0283595.s002.pdf]

S2 Table. Table of missingness, ranges, and medians of social determinant of health features in Add Health.

Features	Missing, *N* (%)	Minimum, value	Maximum, value	Median, value
Lives in one-family house	1 (0.02)	0 *No*	1 *Yes*	1 *Yes*
Frequency of hanging out with friends	6 (0.09)	1 *Not at all*	4 *5 or more times*	3 *3 or 4 times*
Eats no breakfast	8 (0.12)	0 *No*	1 *Yes*	0 *No*
Needed medical attention but did not seek treatment	12 (0.18)	0 *No*	1 *Yes*	0 *No*
Body image	14 (0.22)	1 *Very underweight*	5 *Very overweight*	3 *About the right weight*
Daily hours being online	20 (0.31)	0 hours	99 hours	2 hours
Daily hours watching TV	27 (0.42)	0 hours	99 hours	11 hours
Daily hours driving	28 (0.43)	0 hours	4 hours	1 hour
Employment status	31 (0.48)	0 *No*	1 *Yes*	1 *Yes*
Happy living in their town	32 (0.49)	1 *Not at all*	5 *Very much*	4 *Quite a bit*
Perceived town safety	36 (0.55)	0 *No*	1 *Yes*	1 *Yes*
Number of siblings	47 (0.72)	0 siblings	13 siblings	1 sibling
Desire to attend college	57 (0.88)	1 *Low*	5 *High*	5 *High*
Anticipation of being killed by age 21	59 (0.91)	1 *No chance*	5 *Almost certain*	1 *No chance*
Physical altercations while intoxicated	60 (0.92)	0 *No*	1 *Yes*	0 *No*
STD risk	81 (1.25)	1 *Very high*	5 *No chance*	5 *No chance*
Ever had intercourse	86 (1.32)	0 *No*	1 *Yes*	0 *No*
Being Baptist	139 (2.14)	0 *No*	1 *Yes*	0 *No*
Being Catholic	139 (2.14)	0 *No*	1 *Yes*	0 *No*
Being Christian	139 (2.14)	0 *No*	1 *Yes*	0 *No*
Being non-religious	139 (2.14)	0 *No*	1 *Yes*	0 *No*
English grades	321 (4.94)	1 *A*	4 *D or lower*	2 *B*
Perception that their mother cares	374 (5.75)	1 *Not at all*	5 *Very much*	5 *Very much*
Math grades*	582 (8.95)	1 *A*	4 *D or lower*	2 *B*
Perception that their father cares*	1957 (30.10)	1 *Not at all*	5 *Very much*	5 *Very much*

*Note. *These features contained a high degree of missingness and were removed before further analyses. Perception that their mother cares was retained given it had only marginally unacceptable missingness.*
