# Supplementary material for: Target-based fusion using social determinants of health to enhance suicide prediction with electronic health records
Source: PLoS One. 2023 Apr 26;18(4):e0283595. doi: 10.1371/journal.pone.0283595 (PMC10132649; doi:10.1371/journal.pone.0283595)
Supplement: S3 Table — (PDF) [file pone.0283595.s003.pdf]

S3 Table. Add Health coding form for similarity matching with HIDD.

Features	Variable	Description	Range and coding
Demographic information			
Age	-	Patient age	<u>Coding</u><br>Study year (1/1/1994) – birth date
Birth date item	-	"*What is your birth date? [month and year].*"	
Birth month item	H1GI1M	"*…Month*"	<u>Range</u><br>1 through 12
Birth year item	H1GI1Y	"*…Year*"	<u>Range</u><br>Open-ended numeric value
Gender	-	Patient gender	<u>Coding</u><br>(0) *Female*<br>(1) *Male*
Biological sex item for interviewer	BIO_SEX	"*Interviewer, please confirm that R's sex is (male) female. (Ask if necessary.)*"	<u>Range</u><br>(1) *Male*<br>(2) *Female*
ICD-9 Codes			
042.XX: Human immunodeficiency virus (HIV) disease	-	Mock HIV disease diagnosis	<u>Coding</u><br>(0) *No*: STD item or had intercourse item = 0<br>(1) *Yes*: STD item = 1
STD item	H1CO16D	"*Have you ever been told by a doctor or a nurse that you had... HIV or AIDs*"	<u>Range</u><br>(0) *No*<br>(1) *Yes*
Ever had intercourse item	H1CO1	"*Have you ever had sexual intercourse? When we say sexual intercourse, we mean when a male inserts his penis into a female's vagina.*"	<u>Range</u><br>(0) *No*<br>(1) *Yes*
054.XX: Herpes simplex	-	Mock herpes simplex diagnosis	<u>Coding</u><br>Same as above
STD item	H1CO16E	"*Have you ever been told by a doctor or a nurse that you had... Genital herpes*"	<u>Range</u><br>Same as above

Ever had intercourse item	H1CO1	Same as above	Range<br>Same as above
070.XX: Viral hepatitis	-	Simulated Hepatitis B diagnosis	Coding<br>Same as above
STD item	H1CO16H	“Have you ever been told by a doctor or a nurse that you had... Hepatitis B”	Range<br>Same as above
Ever had intercourse item	H1CO1	Same as above	Range<br>Same as above
078.XX: Other diseases due to viruses and chlamydiae	-	Mock genital warts diagnosis	Coding<br>Same as above
STD item	H1CO16F	“*Have you ever been told by a doctor or a nurse that you had... Genital warts*”	Range<br>Same as above
Ever had intercourse item	H1CO1	Same as above	Range<br>Same as above
079.XX: Viral and chlamydial infection in conditions classified elsewhere and of unspecified site	-	Mock chlamydia diagnosis	Coding<br>Same as above
STD item	H1CO16A	“*Have you ever been told by a doctor or a nurse that you had... Chlamydia*”	Range<br>Same as above
Ever had intercourse item	H1CO1	Same as above	Range<br>Same as above
090.XX: Congenital syphilis	-	Mock syphilis diagnosis	Coding<br>Same as above
STD item	H1CO16B	“*Have you ever been told by a doctor or a nurse that you had... Syphilis*”	Range<br>Same as above
Ever had intercourse item	H1CO1	Same as above	Range<br>Same as above
098.XX: Gonococcal infections	-	Mock gonorrhea diagnosis	Coding<br>Same as above
STD item	H1CO16C	“*Have you ever been told by a doctor or a nurse that you had... Gonorrhea*”	Range<br>Same as above

Ever had intercourse item	H1CO1	Same as above	<u>Range</u><br>Same as above
131.XX: Trichomoniasis	-	Mock trichomoniasis diagnosis	<u>Coding</u><br>Same as above
STD item	H1CO16G	"*Have you ever been told by a doctor or a nurse that you had… Trichomoniasis*"	<u>Range</u><br>Same as above
Ever had intercourse item	H1CO1	Same as above	<u>Range</u><br>Same as above
250.XX: Diabetes mellitus	-	Mock diabetes mellitus diagnosis	<u>Coding</u><br>None
Diabetes item from parent survey	PC49F_1	"*Diabetes. (NAME) has?*"	<u>Range</u><br>(0) *No*<br>(1) *Yes*
278.XX: Overweight, obesity, and hyperalimentation	-	Mock obesity diagnosis	<u>Coding</u><br>Same as above
Obesity item from parent survey	PC49A_1	"*Obesity. (NAME) has?*"	<u>Range</u><br>Same as above
296.XX: Episodic mood disorders	-	Mock depression diagnosis	<u>Coding</u><br>(0) *No*: total score < 16<br>(1) *Yes*: total score >= 16
Center for Epidemiological Studies Depression Scale (CES-D) items	H1FS1 to H1FS19	Total score	<u>Range</u><br>0 through 60
303.XX: Alcohol dependence syndrome	-	Mock alcohol dependence diagnosis	<u>Coding</u><br>(0) *No*: item > 2<br>(1) *Yes*: item <= 2
intoxicated frequency item	H1TO18	"*Over the past 12 months, on how many days have you gotten drunk or 'very, very high' on alcohol?*"	<u>Range</u><br>(1) *Everyday/almost everyday*<br>…<br>(7) *Never*

304.XX: Drug dependence	-	Mock marijuana dependence diagnosis	<u>Coding</u><br>(0) *No*: frequency or ever item = 0<br>(1) *Yes*: frequency item >= 4 days per week
Smoking frequency item	H1TO32	"*During the past 30 days, how many times did you use marijuana?*"	<u>Range</u><br>Open-ended numeric
Ever smoked item	H1TO30	"*How old were you when you tried marijuana for the first time? If you never tried marijuana, enter '0.'*"	<u>Range</u><br>(0) *Never*<br>(1) *1 year old*<br>…<br>(18) *18 years old or older*
305.XX: Nondependent abuse of drugs	-	Mock substance abuse program diagnosis	<u>Coding</u><br>None
Abuse program item	H1HS5	"*In the past year, have you attended a drug abuse or alcohol abuse treatment program?*"	<u>Range</u><br>(0) *No*<br>(1) *Yes*
315.XX: Specific delays in development	-	Mock learning disability diagnosis	<u>Coding</u><br>Same as above
Learning disability item from parent survey	PC38	"*Does (he/she) have a specific learning disability, such as difficulties with attention, dyslexia, or some other reading, spelling, writing, or math disability?*"	<u>Range</u><br>Same as above
346.XX: Migraine	-	Mock migraine diagnosis	<u>Coding</u><br>Same as above
Migraine item from parent survey	PC49B_1	"*Migraine headaches. (NAME) has?*"	<u>Range</u><br>Same as above
477.XX: Allergic rhinitis	-	Mock allergy diagnosis	<u>Coding</u><br>Same as above
Allergy item from parent survey	PC49C_1	"*Allergies or hay fever. (NAME) has?*"	<u>Range</u><br>Same as above

493.XX: Asthma	-	Mock asthma diagnosis	<u>Coding</u><br>Same as above
Asthma item from parent survey	PC49D_1	*"Asthma or emphysema. (NAME) has?"*	<u>Range</u><br>Same as above
706.XX: Diseases of sebaceous glands	-	Mock acne diagnosis	<u>Coding</u><br>(0) *No*: item < 3<br>(1) *Yes*: item >= 3
Skin problem frequency item	PC49D_1	*"Please tell me how often you have had each of the following conditions in the past 12 months. Skin problems, such as itching or pimples?"*	<u>Range</u><br>(0) *Never*<br>…<br>(4) *Everyday*
719.XX: Other and unspecified disorders of joint (includes difficulty walking)	-	Mock walking aid or device diagnosis	<u>Coding</u><br>None
Use of walking aid or device item	H1PL5	*"Do you use a cane, crutches, walker, medically prescribed shoes, wheelchair, or scooter to get around because of a permanent physical condition?"*	<u>Range</u><br>(0) *No*<br>(1) *Yes*
729.XX: Other disorders of soft tissue	-	Mock muscle and joint disorder diagnosis	<u>Coding</u><br>(0) *No*: item < 3<br>(1) *Yes*: item >= 3
Muscle/joint pain frequency item	H1GH15	*"Please tell me how often you have had each of the following conditions in the past 12 months. Aches, pains, or soreness in your muscles or joints"*	<u>Range</u><br>(0) *Never*<br>…<br>(4) *Everyday*
780.XX: General symptoms (includes insomnia)	-	Mock insomnia diagnosis	<u>Coding</u><br>Same as above
Insomnia frequency item	H1GH18	*"Please tell me how often you have had each of the following conditions in the past 12 months. Trouble falling asleep or staying asleep"*	<u>Range</u><br>Same as above

784.XX: Symptoms involving head and neck	-	Mock head pain and symptoms diagnosis	<u>Coding</u><br>Same as above
Headache frequency item	H1GH2	*"Please tell me how often you have had each of the following conditions in the past 12 months. How often have you had a headache?"*	<u>Range</u><br>Same as above
786.XX: Symptoms involving respiratory system and other chest symptoms	-	Mock chest pain and symptoms diagnosis	<u>Coding</u><br>Same as above
Chest pain frequency item	H1GH14	*"Please tell me how often you have had each of the following conditions in the past 12 months. Chest pains"*	<u>Range</u><br>Same as above
788.XX: Symptoms involving urinary system	-	Mock urination pain and symptoms diagnosis	<u>Coding</u><br>Same as above
Urination pain frequency item	H1GH9	*"Please tell me how often you have had each of the following conditions in the past 12 months. Painful or very frequent urination (or peeing)"*	<u>Range</u><br>Same as above
789.XX: Other symptoms involving abdomen and pelvis	-	Mock abdominal pain and symptoms diagnosis	<u>Coding</u><br>Same as above
Stomachache frequency item	H1GH4	*"Please tell me how often you have had each of the following conditions in the past 12 months. A stomach ache or an upset stomach"*	<u>Range</u><br>Same as above
799.XX: Other ill-defined and unknown causes of morbidity and mortality (includes irritability)	-	Mock anger and irritability symptom diagnosis	<u>Coding</u><br>None
Temper item from parent survey	PC32	*"Does (NAME) have a bad temper?"*	<u>Range</u><br>(0) *No*<br>(1) *Yes*
E92X.XX: Other accidents	-	Mock gunshot injury diagnosis	<u>Coding</u><br>(0) *No*: item = 0

			(1) *Yes*: item > 0
Gunshot frequency item	H1FV3	“*During the past 12 months, how often did each of the following things happen? Someone shot you*”	Range<br>(0) *Never*<br>(1) *Once*<br>(2) *More than once*
E96X.XX: Homicide and injury purposely inflicted by other persons	-	Mock stab by weapon injury diagnosis	Coding<br>Same as above
Stabbed by weapon frequency item	H1FV4	“*During the past 12 months, how often did each of the following things happen? Someone cut or stabbed you.*”	Range<br>Same as above
V70.XX: General medical examination	-	Mock general health examination	Coding<br>(0) *No*: item > 1<br>(1) *Yes*: item = 1
Physical exam item	H1GH24	“*When did you last have a physical examination by a doctor or nurse?*”	Range<br>(1) *Less than a year ago*<br>…<br>(4) *Never*
V72.XX: Special investigations and examinations	-	Mock dental health visit	Coding<br>Same as above
Dental health visit item	H1GH25	“*When did you last have a dental examination by a dentist or hygienist?*”	Range<br>Same as above
