# Supplementary material for: Target-based fusion using social determinants of health to enhance suicide prediction with electronic health records
Source: PLoS One. 2023 Apr 26;18(4):e0283595. doi: 10.1371/journal.pone.0283595 (PMC10132649; doi:10.1371/journal.pone.0283595)
Supplement: S1 File — (PDF) [file pone.0283595.s004.pdf]

**S4 File. Similarity matching between HIDD and Add Health data.**

Social determinant of health features were transferred to the HIDD by matching Add Health and HIDD individuals on shared features between datasets. For simplicity, let ($x_i^t$, $y_i^t$) be a feature vector and event value, respectively, for any $i^{th}$ HIDD patient where $i$ = 1, …, $n^t$ the total sample size of the HIDD, and ($x_j^e$, $y_j^e$) the same for any $j^{th}$ Add Health participant where $j$ = 1, …, $n^e$ the total sample size of Add Health. To quantify the similarity between HIDD patients and Add Health participants, Pearson's correlation coefficient $\{r_{ij}\}$ and Manhattan distance $\{d_{ij}\}$ were computed, for $q$ = 1, …, $p$ overlapping features, $i$ = 1, …, $n^t$ and $j$ = 1, …, $n^e$. See *Equations 1* and *2* below, respectively, where $W$ is a designated weight vector. Note, Manhattan distance was modified to describe similarity instead of distance.

[1]

$$r_{ij} = \frac{\frac{\sum_q^p W\left(x_{iq}^t - \frac{\sum_q^p W x_{iq}^t}{\sum_q^p W_q}\right)\left(x_{jq}^e - \frac{\sum_q^p W x_{jq}^e}{\sum_q^p W_q}\right)}{\sum_q^p W_q}}{\left(\frac{\sum_q^p W\left(x_{iq}^t - \frac{\sum_q^p W x_{iq}^t}{\sum_q^p W_q}\right)^2}{\sum_q^p W_q}\right)\left(\frac{\sum_q^p W\left(x_{jq}^e - \frac{\sum_q^p W x_{jq}^e}{\sum_q^p W_q}\right)^2}{\sum_q^p W_q}\right)}$$

[2]

$$d_{ij} = 1 - \frac{\sum_q^p W_q \left|x_{iq}^t - x_{jq}^e\right|}{p}$$

Unweighted (i.e., $W_1$= 1, … ,$W_p$ = 1) and weighted versions of similarity scores were computed within genders (and opposite gender scores were coded as zero), and excluding age. Weights were derived to inform similarity by Add Health suicide risk. Specifically, the identical modelling procedures was followed as in primary analyses (excluding marginal screening), predicting suicide risk by age, gender, and overlapping features. Each ICD-9 feature value in computations was weighted by their exponentiated odds $\beta$ of suicide risk in the model. For any $\beta$ < 1, $\beta_0$, they were inverted to maintain a sense of relative weighting from 1 (no weight) to infinity, where $\beta_0$ = 1/$\beta_0$. Similarity scores were also weighted by age difference. Specifically, a vector of absolute age difference ranks was calculated for each HIDD patient, comparing their age to each Add Health participant, and score-age difference rank products were used for matching.
